# Small intestinal perforation due to a huge gastrointestinal stromal tumor in a kidney transplant recipient: a case report and literature review

**DOI:** 10.1186/s12882-019-1310-5

**Published:** 2019-04-03

**Authors:** Ryohei Takahashi, Kazunobu Shinoda, Takashi Ishida, Yasuo Hamamoto, Shinya Morita, Hirotaka Akita, Sotaro Kitaoka, Satoshi Tamaki, Hiroshi Asanuma, Tadashi Yoshida, Masahiro Jinzaki, Kaori Kameyama, Mototsugu Oya

**Affiliations:** 1 Department of Urology, Keio University School of Medicine, Tokyo, 160-8582 Japan; 2 Department of Nephrology, Toho University Faculty of Medicine, 7-5-23 Omorinishi Ota-ku, Tokyo, 143-0015 Japan; 3 Department of Surgery, Keio University School of Medicine, Tokyo, 160-8582 Japan; 4 Keio Cancer Center, Keio University School of Medicine, Tokyo, 160-8582 Japan; 5 Department of Diagnostic Radiology, Keio University School of Medicine, Tokyo, 160-8582 Japan; 6 Apheresis and Dialysis Center, Keio University School of Medicine, Tokyo, 160-8582 Japan; 7 Department of Diagnostic Pathology, Keio University School of Medicine, Tokyo, 160-8582 Japan

**Keywords:** Gastrointestinal stromal tumor, Spontaneous rupture, Kidney transplant recipient, And imatinib mesylate

## Abstract

**Background:**

Gastrointestinal stromal tumors (GISTs) in transplant recipients are very rare and only a handful of cases have been reported to date. Here we present the first known case of a huge GIST in a kidney transplant recipient with perforation of small intestine.

**Case presentation:**

A 64-year-old male presented at our hospital with right colic pain; he had received an ABO incompatible kidney transplant 6 years earlier and was treated with cyclosporine, mycophenolate mofetil, and methylprednisolone. Radiological evaluation revealed a huge (11 cm in diameter) solitary tumor at the small intestine without distant metastasis. The small intestinal wall at the tumor location was perforated one week after diagnosis and the patient underwent emergency surgery. The pathological findings were compatible with GIST and the tumor consisted of spindle cells with positive staining for KIT, CD34, and DOG1 and negative or weak staining for desmin and S-100 protein. A mutation in exon 11 of the *c-kit* gene was also detected. Cyclosporine was withdrawn and imatinib mesylate (400 mg daily) was introduced. However, thereafter, we needed to decrease the dose at 300 mg daily due to severe hyponatremia. Reduced imatinib treatment was well tolerated and recurrence was not observed for 18 months after surgery.

**Conclusions:**

The occurrence of GISTs in transplant patients is rare, and huge GISTs should be resected immediately after diagnosis because gastrointestinal tract at the tumor site could be perforated. Imatinib treatment is feasible in transplant recipients under immunosuppression, although immunosuppressive drugs metabolized by CYP3A4 should be used at a reduced dosage or withdrawn.

## Background

Malignant diseases occurring after solid organ transplantation are a critical issue that can result in graft loss or patient death with a functioning graft. In cases of a functioning graft, the cumulative incidence rate of malignancy is known to increase with postoperative time. Among such malignancies, gastrointestinal stromal tumors (GISTs) are an especially rare neoplasm and only a handful of cases have been reported in patients following solid organ transplantation [1–8].

The specific pathological features and risk stratification of GISTs in the general population have been actively investigated [9–13]. However, whether the malignant potential of GISTs is higher in patients in an immunosuppressive state remains unknown. In the present report, we describe a huge GIST at the small intestine in a kidney transplant recipient who experienced a perforation of the small intestinal wall at the tumor location shortly after the diagnosis. We also review existing literature on GISTs, which were written in English, in solid organ transplantation recipients and summarized in Tables 1 and 2, including the one in the present report.

**Table 1:** Background of reported cases of patients with GIST after organ transplantation

Case	Author	Year	Transplanted organ	Age/Sex	Primary disease	Time from transplantation to diagnosis (months)	Symptoms/causes at diagnosis	Location	Treatment	Solitary/Multiple
1	Agaimy	2007	Kidney	59/F	Diabetic nephropathy	40	Non-specific abdominal pain	Stomach	Resection	Solitary
2	Agaimy	2007	Kidney	58/F	Glomerulonephritis	96	Non-specific abdominal pain	Small intestine	Resection	Solitary
3	Saidi	2008	Liver	54/M	HCV-HCC	11	Colonoscopy	Ascending colon	Resection	Solitary
4	Camargo	2008	Liver	64/M	HBC-LC, HCC	7	Anal discomfort	Lower rectum	Resection	Solitary
5	Tu	2012	Kidney	57/F	Hypertensive renal failure	6	Non-specific abdominal pain	Pelvic cavity	Resection	Solitary
6	Mulder	2012	Kidney	72/M	Not described	251–262	Upper gastrointestinal bleeding	Stomach	Resection	Solitary
7	Mrzljak	2013	Liver	53/M	Alcoholic LC	Not described	Incidentally at the other operation	Jejunum	Resection	Solitary
8	Cimen	2015	Kidney	46/F	Hypertensive renal failure	216	Ultrasound	Stomach	Resection	Solitary
9	Cheung	2017	Kidney	64/M	Diabetic nephropathy	24	Anemia	Stomach	Resection	Solitary
10	Cheung	2017	Kidney	48/M	FSGS	12	Abdominal mass	Multiple mesentery	Imatinib	Multiple
11	This case	2018	Kidney	64/M	Diabetic nephropathy	72	Right colic pain	Ileum	Resection	Solitary

**Table 2:** Treatment and outcome of reported cases of patients with GIST after organ transplantation

Case	Author	Size (cm)	Nuclear mitotic counts	Fletcher’s criteria	Joensuu’s criteria	Introduction of Imatinib	Immunosuppression before the treatment	Immunosuppression after the treatment	Outcome/Months
1	Agaimy	3.5	< 5/50 HPF	Low	Low	Not described	Not described	Not described	Alive/68
2	Agaimy	23.0	14/50 HPF	High	High	Not described	Not described	Not described	Not described
3	Saidi	2.5	< 5/50 HPF	Low	Low	None	Tac, Azathioprine	Not described	Alive/18
4	Camargo	5.0	5/50 HPF	Intermediate	Low	None	Tac	Not described	Alive/20
5	Tu	4.5	2–3/50 HPF	Low	Low	None	CsA, MMF, Steroid	Steroid withdrawn CsA and MMF were reduced at half dosage	Alive/24
6	Mulder	5.0	> 10/50 HPF	High	High	400 mg/day → 200 mg/day	CsA, Steroid	CsA dosage was reduced from 110 mg daily to 75 mg daily	Recurrence/21 Death/44
7	Mrzljak	1.0	1/50 HPF	Low	Low	None	Tac, MMF	Not described	Death/38 Unknown cause
8	Cimen	15.0	14/50 HPF	High	High	400 mg/day	CsA, Azathioprine, Steroid	CsA trough level at 200–350 μg/L	Alive/12
9	Cheung	3.0	9/50 HPF	High	Intermediate	None	CsA → Tac Azathioprine →MMF, Steroid	Tac trough level at 2.6 μg/L MMF was replaced with Everolimus (trough level at 6.7 μg/L)	Liver metastasis/24 Death/24 Multidrug-resistant bacterial pneumonia
10	Cheung	Not described	Not described	Not described	Not described	400 mg/day	CsA, MMF	CsA withdrawn Sirolimus introduction (trough level at 5.1 μg/L)	Alive/120
11	This case	11.0	20/50 HPF	High	High	400 mg/day → 300 mg/day	CsA, MMF, Steroid	CsA withdrawn	Alive/18

## Case presentation

A 64-year-old male with diabetic nephropathy received an ABO-incompatible kidney transplantation using a donated kidney from his wife in August 2011. Splenectomy was performed one month before the transplant and plasmapheresis was performed three times for the preconditioning treatment. An interleukin-2-receptor monoclonal antibody (basiliximab) was used as an induction immunosuppressant and maintenance immunosuppression included cyclosporine (target trough level, 150–200 ng/mL for the first month after transplant; 100–150 ng/mL for the second month; 50–100 ng/mL from the third month to one year after transplant; and 30–50 ng/mL thereafter), mycophenolate mofetil (1500 mg daily), and methylprednisolone (starting dose of 20 mg daily with a subsequent weekly reduction by 4 mg and maintenance dosage at 4 mg thereafter). His postoperative course was good with no evidence of acute rejection. His serum creatinine level and estimated glomerular filtration rate level were 1.3 ± 0.6 mg/dL and 48 ± 5 mL/min/1.73 m^2^, respectively, at 6 years after transplantation.

**Fig. 1:**
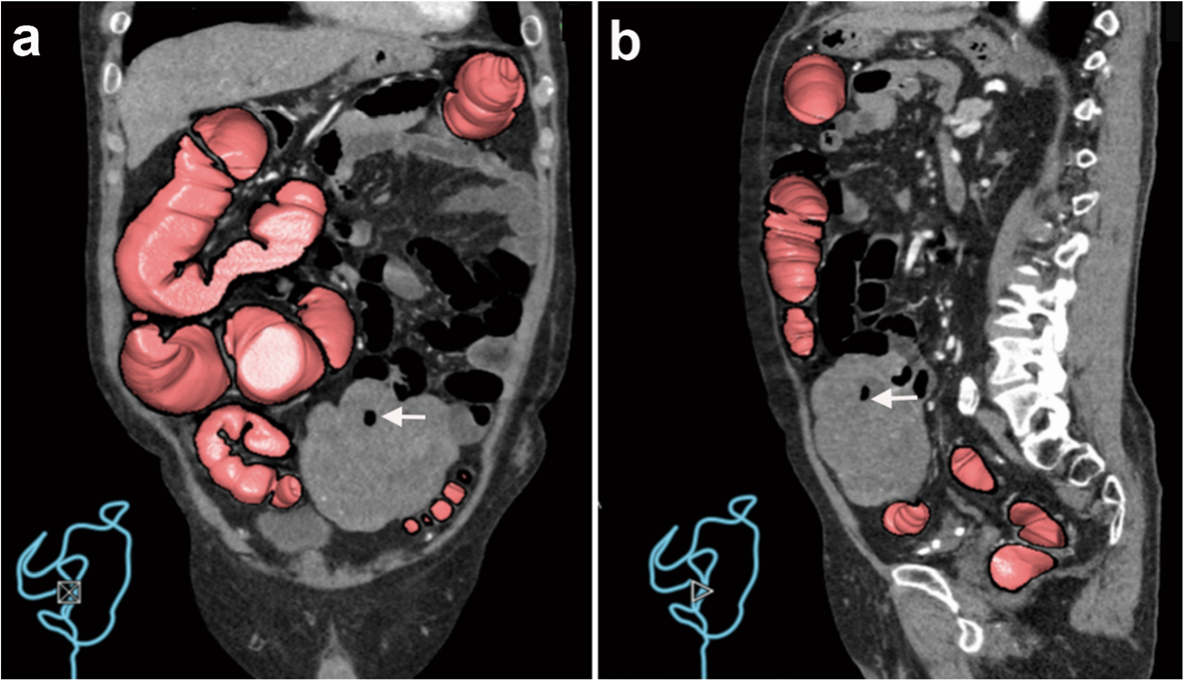
Virtual endoscopic images with multi-planar reconstruction on computed tomography colonography. **a** Coronal and (**b**) sagittal planes show the huge tumor originating from the small intestine and not from the colon. The bowel tract (in pink) represents the colorectum. The arrows represent an air bubble in the tumor

**Fig. 2:**
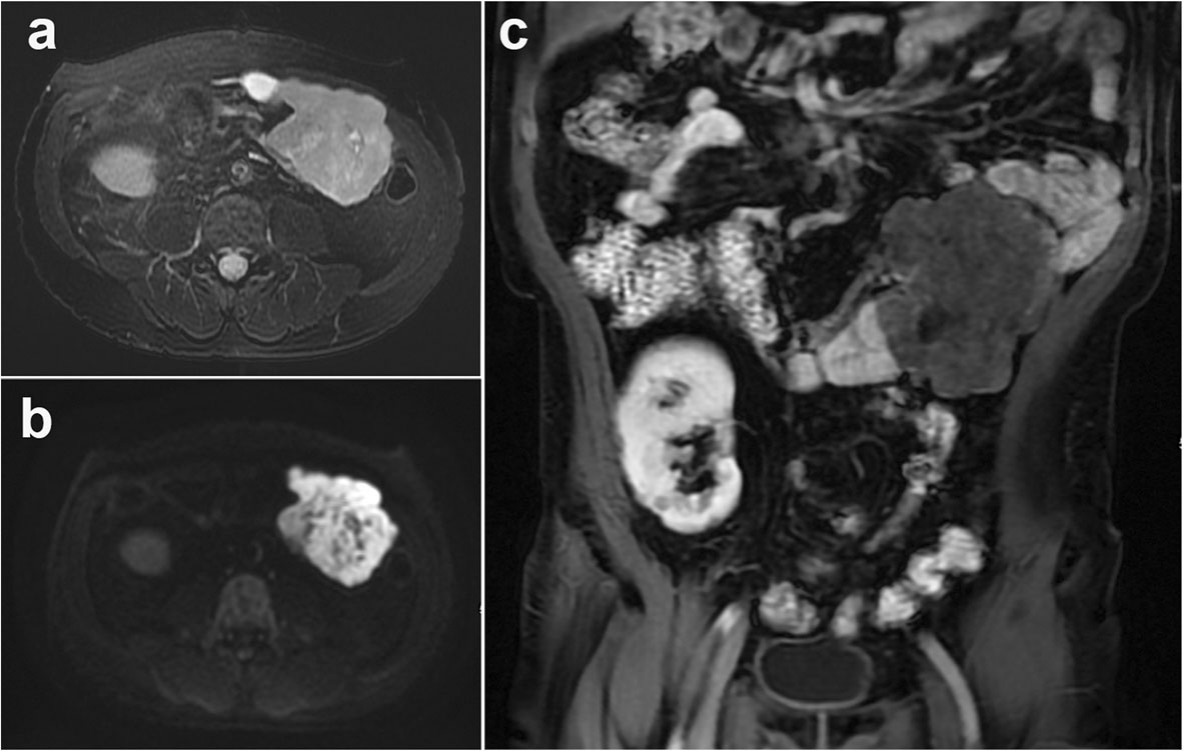
Magnetic resonance imaging scans of the gastrointestinal stromal tumor. **a** The tumor showed moderately high signal intensity on a fat-suppressed T2-weighted image. **b** The tumor clearly showed high signal intensity on a diffusion-weighted image. **c** Contrast-enhanced T1-weighted coronal image with fat suppression showed a weak enhancement in the tumor

He visited our outpatient department of surgery in July 2017 (72 months after the transplant), suffering from right colic pain, abdominal distention, and diarrhea. The initial non-contrast-enhanced computed tomography (CT) scan revealed a solitary tumor with a diameter of 11 cm in his lower abdomen. CT colonography was performed to determine the tumor location, which revealed an origin from the small intestine, not the colon or the sigmoid rectum, expanding outside the lumen (Fig. 1). The tumor was well circumscribed with a smooth boundary and lobulated contour. There were relatively low attenuation areas in the tumor, which were suspected to correspond to the areas of necrotic degeneration. Moreover, a trapped air bubble was detected in the tumor and mucosal ulcer formation at the tumor site was suspected. Distant metastasis was not observed on CT. Magnetic resonance imaging revealed a moderately high signal intensity on fat-suppressed T2-weighed images and a clear high signal intensity on diffusion-weighted images, which suggested the tumor had malignant potential (Fig. 2). The preoperative differential diagnosis was GIST or post-transplant lymphoproliferative disorder of the small intestine. Resection of the tumor was planned several weeks later because operation room schedule was very tight at that time.

**Fig. 3:**
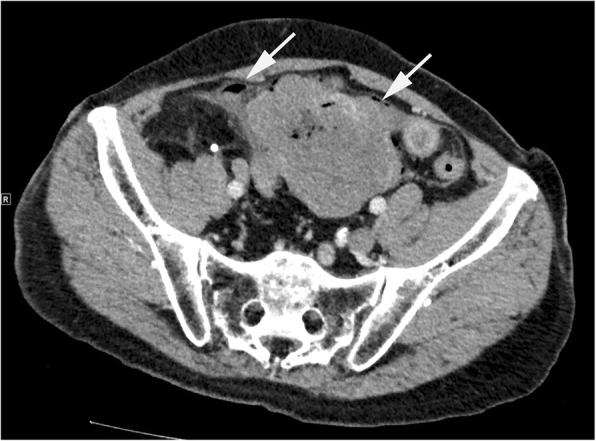
Emergent computed tomography images on the day of the operation. Arrows represent free air close to the tumor

**Fig. 4:**
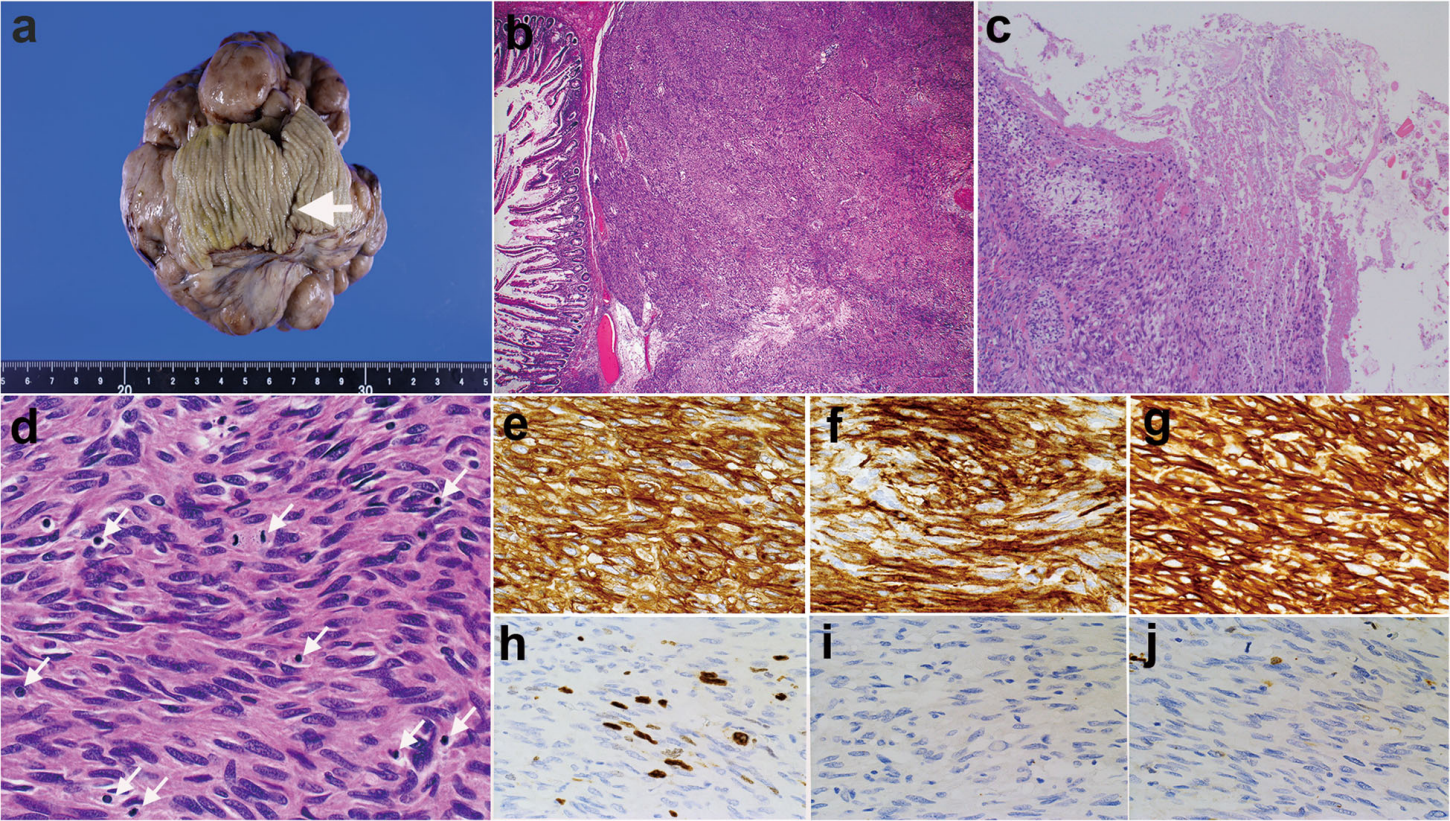
Pathological images of the gastrointestinal stromal tumor. **a** Macroscopic finding of the tumor represents that this was a multinodular tumor continuous to the intestinal wall. The arrow represents the perforated hole. **b** The tumor was located just under the muscularis mucosa, and the boundary was clear. [Hematoxylin-Eosin (HE) × 4]. **c** The central part of the tumor fell into necrosis and became cystic. **d** Tumor cells were spindle shaped cells with rodlike hyperchromatic nuclei. The arrows represent the cells during mitosis (HE × 40). The tumor cells were strongly positive for KIT (**e**), CD34 (**f**), and DOG1 (**g**) stainings. **h** The positive ratio of Ki-67 staining was 20%. The staining for desmin was negative (**i**) and the staining for S-100 protein was weak (**j**)

One week after diagnosis, the patient visited the emergency room suffering from acute onset of abdominal pain. Emergent CT revealed free air around the tumor and in the upper peritoneal cavity, suggesting perforation of the small intestine (Fig. 3). Emergent laparotomy was performed and revealed that small intestinal perforation had occurred due to tumor necrosis on the luminal side. Cytological examination of ascites, which were collected during the operation, showed no malignant cells. Resection of the tumor and intestinal anastomosis was performed simultaneously. The patient’s postoperative course was good without any comorbidity. The pathological findings showed a perforation hole, 2 mm in diameter, at the intestinal wall above the tumor (Fig. 4a). The tumor had a clear boundary and grew nodularly from just below the muscularis mucosa towards the abdominal cavity (Fig. 4b). In the center of the tumor there was a cavity due to coagulative necrosis, which resulted in tumor rupture (Fig. 4c). The tumor consisted of a bundle of spindle cells that were strongly positive for KIT, CD-34, and discovered on gastrointestinal stromal tumor 1 (DOG1); the positive ratio of Ki-67 was 20% (Fig. 4d-h). The nuclear mitotic count was 20/50 in high power fields. Negative staining of desmin and weak staining for S-100 protein excluded the possibility of a leiomyoma or schwannoma (Fig. 4i, j). These pathological findings supported a diagnosis of GIST. Risk classification of the tumor was classified as high-risk. Genetic screening revealed a mutation in exon 11 of *c-kit* and the deletion of two amino acids (Tyr553Trp557del).

Imatinib mesylate at a dose of 400 mg daily was introduced 2 months after the operation as an adjuvant therapy due to the tumor’s high malignant potential, its size (> 5 cm), and the mitotic count (20/50 in high power fields) [12, 13]. We needed to decrease the dose at 300 mg daily a month after the introduction of imatinib because the patient experienced severe hyponatremia (112.7 mmol/L). Although the cyclosporine trough level was relatively low (30–50 ng/mL) before and after the operation, we withdrew cyclosporine 2 months after the operation without tapering off. Therefore, maintenance immunosuppression comprised mycophenolate mofetil (1500 mg daily) and methylprednisolone (4 mg daily). The patient tolerated reduced imatinib treatment well and his kidney function was well preserved without any evidence of rejection. At follow-up 18 months after the operation, there was no recurrence of the tumor.

## Discussion and conclusions

The present case described a huge GIST with perforated small intestine in a kidney transplant recipient in a long-term immunocompromised state due to the combination of immunosuppressive drugs. To the best of our knowledge, this is the first reported case of a GIST with perforated small intestine at the site in a kidney transplant recipient. Because a huge GIST can perforate the gastrointestinal tract, immediate surgical resection, if appropriate, should be considered.

GISTs have unique characteristics that are useful when differentiating from other mesenchymal tumors, e.g.*,* leiomyoma, leiomyosarcoma, schwannoma, desmoid tumor, inflammatory myofibroblastic tumor, or solitary fibrous tumor. In general, GISTs originate in the submucosal layer of the gastrointestinal tract and grow extraluminally or intraluminally. More than 90% of these neoplasms are associated with mutations of the proto-oncogene *c-kit* where its encoding protein KIT, a type III receptor tyrosine kinase, is constitutively expressed without stimulation of its ligand, the stem cell factor [9]. Strong positivity of KIT or CD34 is a typical pathological feature of GISTs [10]. DOG1 was recently reported as a specific GIST marker and ubiquitous expression of DOG1 was observed in patients with GISTs [11]. Scarce positivity of desmin and S-100 protein also help differentiate GISTs from other mesenchymal tumors [10]. Interestingly, only subpopulations of GISTs have malignant potential and several risk stratifications have been advocated according to tumor size, site of organ, and number of mitoses [12, 13]. Tumor size is very important; in the present case, the tumor diameter was 11 cm and the small intestinal wall at the site of the tumor was perforated before the scheduled operation.

To review the previously reported cases of GISTs in transplant recipients, we used the keywords “gastrointestinal stromal tumor,” “transplantation,” OR “transplant” to search PubMed and Web of Science for English language reports. We also checked articles in the reference lists of these case reports [1–8]. Tables 1 and 2 summarize these patients, including the one in the present case.

Some risk criteria of GISTs according to size, nuclear mitotic counts, and tumor location have been advocated in the previous papers [12, 13]. The search revealed only five high-risk (early tumor recurrence of metastasis) cases of 11 total cases according to Fletcher’s criteria [12] or four high-risk cases of 11 total cases according to Joensuu’s criteria [13], including this case, which suggests that immunosuppressed patients are not necessarily at a high risk of GISTs [13] (Table 2). Adjuvant therapy with imatinib mesylate, a tyrosine kinase inhibitor, at a dose of 400 mg daily for three years is recommended for high-risk patients [14]. Precise genetic analysis of *c-kit* is also useful when considering the efficacy of imatinib treatment, and a good response to imatinib treatment in GIST patients is associated with mutations in exon 11 [15].

Another concern is the interaction between imatinib mesylate and immunosuppressive drugs and how to modify immunosuppression during treatment for GISTs. Imatinib mesylate is mainly metabolized by cytochrome P450 3A4 (CYP3A4) in the liver, as is cyclosporine [16]. The concentration of cyclosporine can potentially increase with the combined use of imatinib mesylate and cyclosporine because metabolism of cyclosporine may be competitively inhibited.

Although there was no direct evidence about the relationship between cyclosporine use and GISTs, the etiology of some type of malignancy in patients treated with calcineurin inhibitors (e.g. cyclosporine and tacrolimus) was suggested as the inhibition of tumor cell-specific cytotoxic T cells [17]. Interestingly, Rusakiewicz et al. [18] reported that the number of infiltrating CD3+ T cells was inversely correlated with tumor size of localized GISTs. GIST may grow larger if the number of T cells is low and tumor cell-specific cytotoxic T cells may thus actively inhibit GIST growth. When possible, cyclosporine should be used at a lower dosage or withdrawn during treatment of GISTs. In the literature review, cyclosporine or tacrolimus were reduced or withdrawn in five in six cases of transplant patients with GISTs in whom immunosuppression modification was described (Table 2). By contrast, inhibitors of mammalian target of rapamycin (mTOR), such as everolimus, may be beneficial for treatment of GISTs in transplant patients. The mTOR pathway is critical for lymphocyte activation as well as angiogenesis, which are critical for cell growth and metastasis [19]. Combination use of everolimus and imatinib mesylate was well tolerated and a synergistic antiproliferative effect was observed in imatinib-resistant GIST cell lines [20]. The introduction of mTOR inhibitors was conducted in two cases (Table 2).

The limitation of this report is that we did not monitor trough levels of imatinib mesylate after reducing its dose. It was reported that bioavailability of imatinib decreased 30% from the baseline about 3 months after the introduction [21]. We reduced the dose of imatinib (400 mg daily to 300 mg daily) due to grade 3 hyponatremia, although we did not evaluate whether the dose of 300 mg daily is optimum for the patient. Monitoring of imatinib trough levels may be required to confirm whether individual bioavailability is within optimum range.

In conclusion, because a huge GIST can perforate the gastrointestinal tract in transplant recipients, they should be resected immediately after diagnosis. Imatinib treatment is feasible in transplant recipients under immunosuppression, although immunosuppressive drugs metabolized by CYP3A4 should be used at a reduced dosage or withdrawn. Modifications to combinations of immunosuppressive drugs should also be considered due to their pro−/anti-tumor effects.

## Abbreviations

CT: Computed tomography
CYP3A4: cytochrome P450 3A4
DOG1: Discovered on gastrointestinal stromal tumor 1
GISTs: Gastrointestinal stromal tumors
mTOR: Mammalian target of rapamycin
